# Tracking the Development of Muscular Myoglobin Stores in Mysticete Calves

**DOI:** 10.1371/journal.pone.0145893

**Published:** 2016-01-20

**Authors:** Rachel Cartwright, Cori Newton, Kristi M. West, Jim Rice, Misty Niemeyer, Kathryn Burek, Andrew Wilson, Alison N. Wall, Jean Remonida-Bennett, Areli Tejeda, Sarah Messi, Lila Marcial-Hernandez

**Affiliations:** 1 California State University Channel Islands, Camarillo, California, United States of America; 2 The Keiki Kohola Project, Lahaina, Hawaii, United States of America; 3 Hawaii Pacific University Stranding Program, College of Natural and Computational Sciences, Hawaii Pacific University, Kaneohe, Hawaii, United States of America; 4 Oregon Marine Mammal Stranding Network, Marine Mammal Institute, Oregon State University, Newport, Oregon, United States of America; 5 International Fund for Animal Welfare, Yarmouth Port, Massachusetts, United States of America; 6 Alaska Veterinary Pathology Services, Eagle River, Alaska, United States of America; Rutgers University -New Jersey Medical School, UNITED STATES

## Abstract

For marine mammals, the ability to tolerate apnea and make extended dives is a defining adaptive trait, facilitating the exploitation of marine food resources. Elevated levels of myoglobin within the muscles are a consistent hallmark of this trait, allowing oxygen collected at the surface to be stored in the muscles and subsequently used to support extended dives. In mysticetes, the largest of marine predators, details on muscular myoglobin levels are limited. The developmental trajectory of muscular myoglobin stores has yet to be documented and any physiological links between early behavior and the development of muscular myoglobin stores remain unknown. In this study, we used muscle tissue samples from stranded mysticetes to investigate these issues. Samples from three different age cohorts and three species of mysticetes were included (total sample size = 18). Results indicate that in mysticete calves, muscle myoglobin stores comprise only a small percentage (17–23%) of conspecific adult myoglobin complements. Development of elevated myoglobin levels is protracted over the course of extended maturation in mysticetes. Additionally, comparisons of myoglobin levels between and within muscles, along with details of interspecific differences in rates of accumulation of myoglobin in very young mysticetes, suggest that levels of exercise may influence the rate of development of myoglobin stores in young mysticetes. This new information infers a close interplay between the physiology, ontogeny and early life history of young mysticetes and provides new insight into the pressures that may shape adaptive strategies in migratory mysticetes. Furthermore, the study highlights the vulnerability of specific age cohorts to impending changes in the availability of foraging habitat and marine resources.

## Introduction

In marine mammals, the ability to tolerate apnea for extended periods is a highly adaptive trait, primarily facilitating foraging within the marine realm[1]. Elevated levels of muscular myoglobin characterize this trait [2]: Myoglobin (Mb), a hemo-protein found within muscle cells, essentially allows for the storage of oxygen that can be used to maintain aerobic respiration and extend aerobic diving capacity. Typically, muscular Mb levels in marine mammals are between 10 and 20 times greater than their terrestrial counterparts [2,3]. Within marine mammals, increasing muscular Mb levels closely track the extension of aerobic dive limits [4] and increase as individuals mature [5–9]. Elevated muscular Mb levels generally comprise one element within a diverse suite of physiological, morphological or behavioral adaptations that facilitate the extension of the dive capacity in marine mammals. Notwithstanding, high Mb levels are a consistent hallmark of the ability to maintain extended aerobic dives [10] and consequently, increases in muscular Mb comprise a key component in the respiratory ontogeny of marine mammals.

To date, the majority of work documenting respiratory ontogeny and the development of muscular Mb in marine mammals has focused on pinnipeds, perhaps reflecting the relative ease of access to younger animals during land-based periods of early development. These studies document a high degree of temporal variability: Periods of rapid increase in muscular Mb levels may occur during the nursing period (e.g. Steller sea lions (*Eumetopias jubatus*); [8]) or during the post weaning fast (e.g. Northern elephant seals (*Mirounga angustirostris*); [11]). Alternatively, Mb increases may be delayed until the onset of foraging (e.g. harbor seals (*Phoca vitulina*); [12]) or until later periods of post-weaning activity (e.g. subantarctic fur seals (*Arctocephalus tropicalis*); [13]). Notably, there are also some exceptions to the typical trajectory of continually increasing Mb levels; for example, in Weddell seals (*Leptonychotes weddellii*), Mb levels peak during the juvenile stage, then subsequently drop to slightly lower levels in mature adults [9].

Within cetacea, documentation of the physiological aspects of respiratory ontogeny is currently limited to a selection of small odontocetes, specifically Fraser’s dolphin (*Lagenodelphis hosei*) [5], spinner dolphin (*Stenella longirostris*) [5], bottlenose dolphin (*Tursiops truncatus)* [7,14] and harbor porpoise (*Phocoena phocoena*) [15]. In these examples, while increasing respiratory capacity and concurrent increases in muscular Mb track increasing age, interspecific variability is equally as pronounced as seen in pinnipeds. For example in harbor porpoise, neonate (estimated age < 2 weeks) muscular Mb levels comprise 50% of adult levels and calves attain adult Mb levels by age 9 to 10 months. By comparison, in bottlenose dolphin, neonate Mb levels comprise around 10% of adult levels and do not reach adult levels until 1.5 years of age [15]. For mysticetes, reports on the physiological ontogeny of the respiratory capacity are sparse. Details of muscular Mb levels for immature age classes of mysticetes are limited to 2 examples, both of which provide details of muscular Mb levels for immature gray whales (*Eschrichtius robustus*) [3,6]. Given the high degree of variation reported for both pinnipeds and odontocetes, differences in development trajectories between different mysticete species likely exist, but remain undocumented to date.

At the molecular level, the underlying biochemical mechanisms that control production of myoglobin within muscle tissue have been studied extensively. Numerous studies have detected interplay between exercise, hypoxia and increased myoglobin production (e.g. [16–23]). Recent tissue-based investigations have provided a detailed description of the biochemical pathway involved. Essentially, during muscular activity calcium is released from within the sarcoplasmic reticulum into the cytosol of the muscle cell. This activates the enzyme calcineurin, causing dephosphorylation of NFAT (nuclear factor of activated T-cells), which in turn translocates into the nucleus of the cell where it promotes expression of myoglobin [24,25]. Hypoxia alone actually impedes production of Mb in muscle cells [24], however in combination with exercise, these two stimuli are associated with increased rates of production of Mb [24,26]. The availability of lipids, acquired during nursing [27] or directly from the diet [28], may also augment the expression of Mb. Comparatively, hypoxia combined with lipid supplementation leads to modest increases in Mb [26]. Exercise then acts as a secondary stimulus, leading to the elevated levels of muscular Mb that characterize marine mammals [26,29].

Live animal studies provide support for these conclusions, although notable interspecific variability within this general framework persists. For example Geiseler et al. [30], comparing rates of increase in muscular Mb levels in active vs. sedentary hooded seals, (*Cystophora cristata*) during early ontogeny, demonstrated that active, freely diving young seals exhibited faster rates of increase of muscular Mb compared to pups initially raised in a sedentary setting. The active pups also exhibited ultimately higher adult Mb levels. Similarly, Ponganis et al. [31] documented differences in levels of muscular Mb in captive vs. wild emperor penguins (*Aptenodytes forster*). In this study, animals raised in captivity, where free-diving was restrained and exposure to hypoxia was limited, exhibited lower adult levels of muscular Mb in comparison to their free-diving, unrestrained wild counterparts. In contrast, Noren et al. [32] recorded moderate increases in muscular Mb levels in young gray seals during the post weaning fast, when seal pup activity is low and pups are not exposed to hypoxia. However pups do have access to a ready supply of lipids during this time, sourced from their lipid-rich blubber, which is laid down during the nursing period. Finally, Noren et al. [15] documented a rapid rise in Mb in very young harbor porpoise, wherein neonate calves achieved 50% of adult levels of Mb within two weeks of parturition. During this period young harbor porpoise would be exposed to hypoxia and they would receive lipid supplementation of the diet through nursing. Additionally, they would be exposed to consistent and sustained exercise, as neonates swim continually alongside their fast-moving mothers [33].

A comparative review of the dynamics of the development of muscular myoglobin stores in mysticetes has the potential to contribute to these investigations and extend our understanding of the ontogeny of the respiratory capacity across the range of marine mammals. In this study, we focus on three species of mysticetes; specifically minke (*Balaenoptera acutorostrata)*, humpback (*Megaptera novaeangliae*) and gray (*Eschrichtius robustus*) whales. All three are migratory capital breeders, making extensive forays between high latitude feeding regions and low latitude breeding regions [34–36]. For all three species, the ontogeny of the respiratory capacity is a vital component of their life history, influencing predation avoidance [37], costs of travel [38] and foraging success of the mother and offspring pair [39–42]. Here, we provide updated and refined estimates of adult Mb levels for the three focal species and describe the trajectories for the development of muscular Mb in these species. We compare levels of Mb within and between muscles, as these details may provide insight into the potential role of exercise in the development of muscular Mb in these species. Finally, we review changes in muscular Mb in the youngest mysticetes, and compare these changes to the differing life histories and early behavior in these three species of mysticetes.

## Method

### Ethics statement

Animal tissues were provided by member organizations within the National Marine Mammal Stranding Network. The work of these groups is overseen and approved by the Office of Protected Resources, National Oceanic and Atmospheric Administration; marine mammal parts are collected under the authority of a NMFS Stranding Agreement issued to each of the co-operating organizations. The laboratory analysis of the tissues was conducted under a Marine Mammal Parts Handling Authorization, provided by regional offices of National Marine Fisheries Service. After reviewing the planned study, each regional office provided a letter of authorization that allowed the shipment of tissues to the lab. As this prior review had been conducted by experts in this field further ethical review by the co-operating institution, California State University Channel Islands, was not required.

### Tissue samples

**Fig 1:**
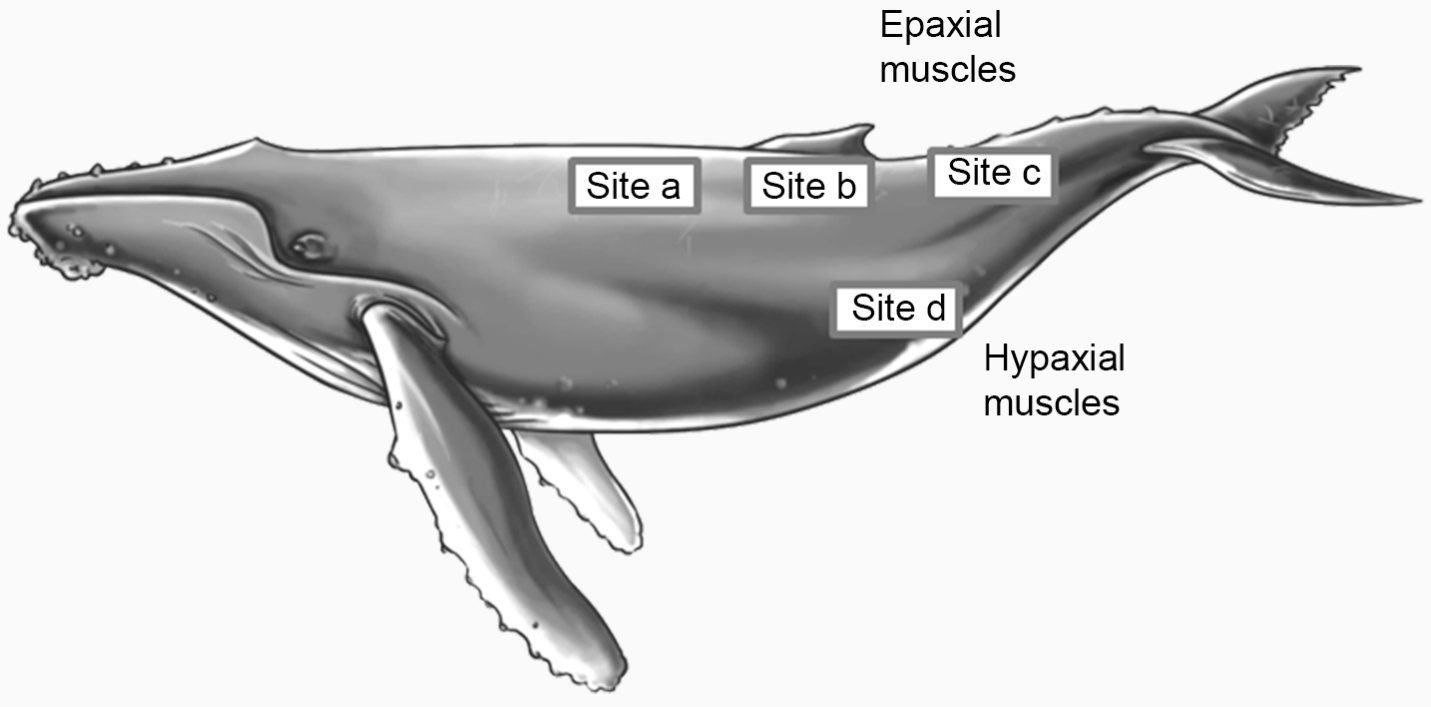
Sampling sites used for the provision of muscle tissue samples from mysticetes. (based on Polasek and Davis [43]). Picture credit: Yvette Hansen.

The Hawaii Pacific University Stranding Program (HPUSP), the Oregon Marine Mammal Stranding Network (OMMSN) the International Fund for Animal Welfare (IFAW) and the Alaska Stranding Network (ASN) provided tissue samples from stranded, deceased animals for this study. Muscle samples were taken from the *longissimus dorsi*, from directly below the dorsal fin (see Fig 1; site b—adapted from [43]). Although details were not always available, the depth of the sample and the precise location of the sampling site were noted whenever possible. For some immature humpback whales (n = 5), samples included the entire longitudinal muscle core at site b, allowing identification of inner and outer portions of the muscle. Additionally, for 3 minke whales and 1 humpback whale calf, samples from the same animal were obtained for a range of positions along the epaxial and hypaxial portions of the major swimming muscles (see Fig 1; sites a—d). All animals were reported as Smithsonian code state 2 or better at the time of the necropsy. Details of dates for each stranding, including causes of death, are provided in S1 Table.

Samples were frozen on collection and stored at -80°C until analysis. Tissue samples were obtained from 18 different whales. Age classes were estimated based on 1) body length details in comparison to published growth curves and / or age-length records (for minke whales; [44], for humpback whales;[45] and for gray whales;[46]), and 2) through the interpretation of field records such as time and location of stranding (See S1 Table).

### Myoglobin Concentration Analysis

Myoglobin levels were assessed following established protocols [3,47]. One gram samples were minced, homogenized in 19.25 ml of 0.04M phosphate buffer solution (pH6.6), sonicated for 30 seconds (using a Power Gen 125 sonicator; Fisher Scientific, Pittsburgh, PA, USA) and centrifuged at 13,000g for 1 hour 15 minutes. The supernatant was collected and bubbled with carbon monoxide for 8 minutes, with 0.02g of sodium thiosulphate added at the mid-point. Absorbance was measured at two key wavelengths, 536 nm and 568nm on a light spectrophotometer (Genesys 10V light spectrophotometer; Thermo Fisher Scientific, Waltham, MA, USA). Reynafarje’s equation was applied to estimate Mb concentration based on the differences in absorbance at the two key wavelengths. All assays were run in triplicate, samples of harbor porpoise muscle tissue were included in each run to verify consistency and estimate the precision of the assay protocol and lyophilized horse myoglobin (Sigma, MD) was used as an additional control to confirm the reliability of the protocol.

### Data Analysis

Mean estimates of myoglobin levels were compiled for each specific age and / or species of whale, for tissues from different muscle sites and from different portions of the muscle. Where tissues were obtained from multiple muscle sites, values obtained for site b (see Fig 1) were used in comparative analyses. As site b is the standard sampling site typically used in field necropsies, this could potentially reduce the impact of differing muscle sites where the sample site was unknown. Where samples were obtained from different depths of the muscle at site b, mean values for all samples from different depths at site b were used. Values for myoglobin levels are reported throughout in milligrams per gram of thawed tissue (mg Mb g^-1^) ± 1 standard error of the mean (S.E.) or ± 1 standard deviation (S.D.) as indicated.

Standard parametric and non-parametric tests were applied using SPSS software to detect significant differences between species, age groups and between different muscle sites, with the level of significance set at 0.05. General linear models (GLM) models were used to detect interactions between factors, and log transformations were used where data was non-normal or variances were unequal. To compensate as much as possible for the small sample sizes in this study, available data from the literature was incorporated into the data analysis wherever possible, and inferential analysis as well as hypothesis based testing was incorporated into data analysis and interpretation.

## Results

### 1. Developmental changes in muscular myoglobin levels

**Fig 2:**
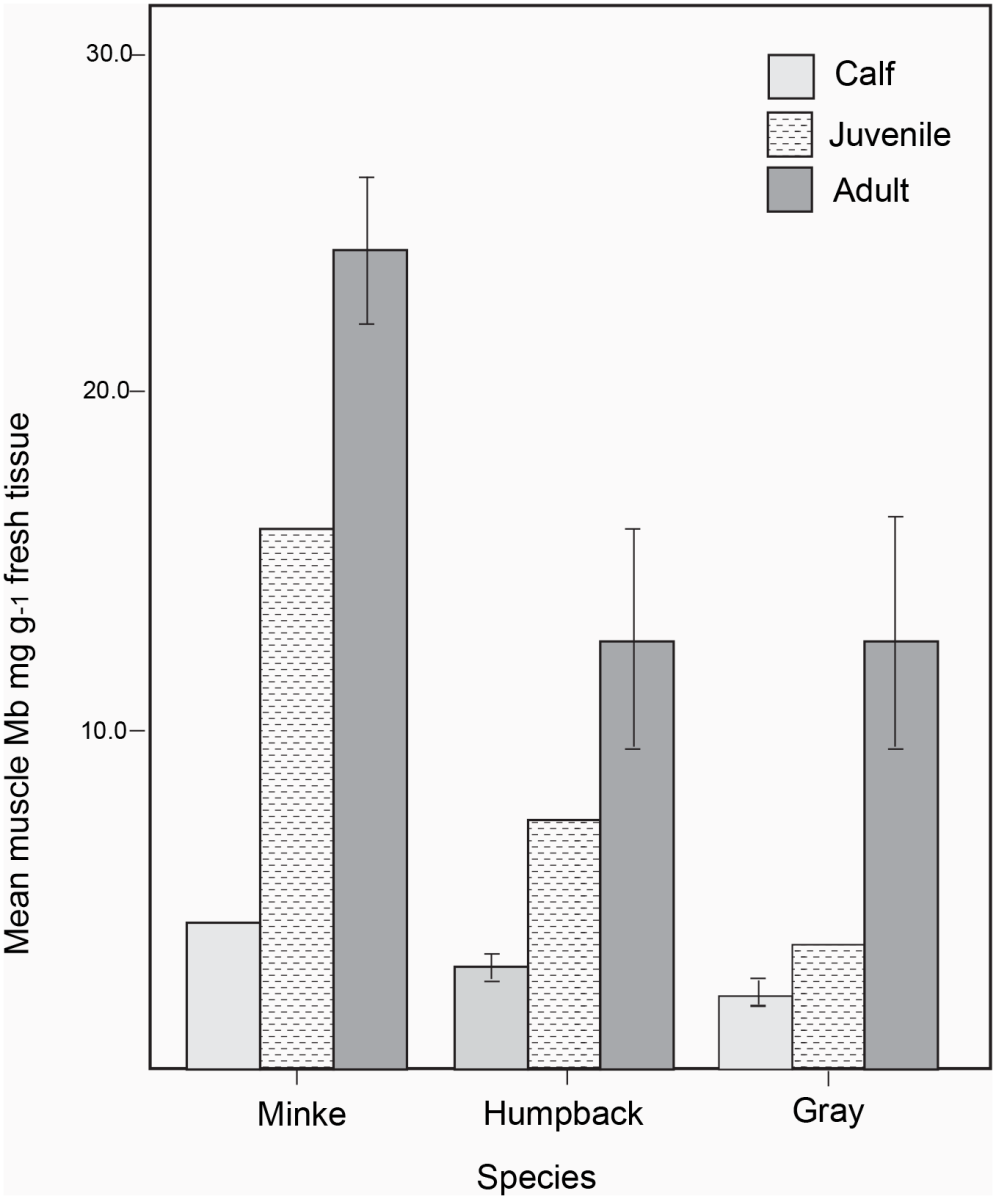
The development trajectory of muscular Mb levels in three species of mysticetes. Differences between age classes and between species were significant (GLM; for age class ANOVA F_2_ = 25.532, p = 0.000, for species ANOVA F _2_ = 5.671, p = 0.018). Error bars indicate +/- 1 S.E.

Looking collectively at the indications from this study, muscular Mb levels increased with age in each of the three species of mysticetes included here (Table 1, Fig 2). Using data from this study alone (n = 18 whales), in minke whales, calf Mb levels represented 17.0% of the mean adult levels, in humpback whales, mean calf levels represented 31.3% of adult levels and in gray whales, mean calf levels represented 21.2% of adult levels. When data available in the literature are included (n = 3; see Table 1), values for minke whales remain unchanged, humpback whale calf levels represented 22.8% of adult levels and gray whale calf levels represented 17.3% of adult levels. Using all 21 samples (i.e. those obtained from this study and from the literature), a GLM was constructed, incorporating muscular Mb as the dependent, and age class and species as fixed factors. All calf data for each species were compiled into a single age cohort (calves). Mb levels were log transformed to adjust for unequal variances in the raw data. The model indicated that differences according to age and species were significant (GLM; for age class ANOVA F_2_ = 25.532, p = 0.000, for species ANOVA F_2_ = 5.671, p = 0.018). Post-hoc tests indicated that significant differences according to age were between calf vs. adult age groups and calf vs. juveniles, while differences between juveniles and adults were marginally non-significant (Tukey test; p < 0.001, p = 0.005 and p = 0.068 respectively). Significant differences between species lay principally between minke vs. humpback whales and minke vs. gray whales (Tukey; p = 0.001 in both cases), while differences between humpback and gray whales were not significant (Tukey; p = 0.930). The GLM indicated that the interaction between age and species was not significant (ANOVA F_4_ = 0.446, p = 0.774). Based on values obtained for the control tissue used (harbor porpoise samples taken from site b), estimated precision was 97.4%.

**Table 1:** Muscular Mb levels according to age class for three species of mysticetes.

Species	Age class	Estimated age	Sample size (N)	Mean muscle Mb (mg Mb g^-1^ tissue) (where n = 2; range, where n>2; +/- 1 S.D.)	Source
Minke Whale *Balaenoptera acutorostrata*					
	Calves	Neonate ^a^	1	4.2	This study
	Juvenile	~ 1 year ^a^	1	14.6	This study
	Adults		2	24.2 (22.1–26.4)	This study
Humpback whale *Megaptera novaeangliae*					
	Calves	Neonate ^b^	1	1.0	This study
		Young calves ^b^	5	3.0 ± 0.4	This study
		Migrating calves ^b^	1	4.6	This study
	Juvenile	~ 2–3 year ^b^	1	7.2	This study
	Adults		2	12.6 (9.4–15.9)	Taken from [48](1) This study (1)
Gray whale *Eschrichtius robustus*					
	Calves	Neonate ^c^	2	1.7 (1.3–2.2)	Taken from [6](1) Taken from [3](1)
		Young calves ^c^	1	2.2	This study
		Migrating calves ^c^	1	3.2	This study
	Juvenile	~ 1 year ^c^	1	3.6	This study
	Adults		2	12.7 (9.2–16.3)	This study

Estimated ages: Neonate age < 2 weeks, young calves between 2 weeks to 3 months and stranded in breeding areas, migrating calves between 3-to 5 months and stranded in migratory corridors. Age estimations based on growth curves described by ^a^ [44], ^b^ [45] and ^c^ [46], along with additional information from the field site of the necropsy. See S1 Table for full details including body lengths for individual specimens and S2 Table for details on literature-sourced data included in the analysis.

### 2. Differences between muscle sites

Comparing Mb levels pairwise between epaxial and hypaxial muscles for samples taken from the same animal, levels were consistently higher in epaxial muscles (Friedman’s test; X^2^ = 7.800, d.f. = 3, p = 0.05; see Table 2). Differences were more pronounced in the younger animals; for the humpback calf and the juvenile minke whale, hypaxial muscle Mb levels represented 59% and 46% of the mean epaxial muscle Mb levels respectively, while in the two adult minke whales, hypaxial muscle Mb levels represented 92% of the mean for epaxial muscle Mb levels.

**Table 2:** Differences in myoglobin levels between epaxial and hypaxial muscles in mysticete whales at different age stages.

Species	Life history stage	Specimen	Muscle sample site	Epaxial muscle Mb (mg Mb g^-1^ tissue)	Hypaxial muscle Mb (mg Mb g^-1^ tissue)
Humpback	Calf	KW2008-003	a	2.7*	
			b	3.8	
			c	2.2*	
			d		1.7
Minke	Juvenile	IFAW 130ba	a	15.4	
			b	15.9	
			c	14.6	
			d		6.9
Minke	Adult	IFAW 131ba	a	19.4	
			b	19.6	
			c	22.0	
			d		17.6
Minke	Adult	IFAW 100ba	a	24.4	
			b	26.4	
			c	21.7	
			d		23.7

Across all samples, differences between epaxial and hypaxial muscles were marginally significant (Friedman’s test; X^2^ = 7.800, d.f. = 3, p = 0.05). Comparing differences at the individual level, differences were much more pronounced in smaller (younger) animals.

* Samples taken from the lower portions of the epaxial muscle, closer to the midline.

### 3. Differences according to depth

Comparing muscle samples taken from different depths within the same muscle (epaxial; site b), muscular Mb levels were consistently higher in the inner portions of the muscle (paired t test; t = 4.020, d.f. = 4, p = 0.016; Table 3). Percentage increases between outer and inner portions were highly variable, ranging from 13% to a more than 100% increase. Differences were more pronounced as levels of Mb increased. It should be noted that all five samples included in the statistical analysis came from humpback whale calves.

**Table 3:** Differences in myoglobin levels between inner and outer portions of the major swimming muscles.

Species	Life history stage	Specimen	Inner swimming muscle (mg Mb g^-1^ tissue)	Outer swimming muscle (mg Mb g^-1^ tissue)	Difference(Inner: outer) (mg Mb g^-1^ tissue)
Humpback	Calf	KW20130012	1.1	1.0	+0.1
		KW20130010	2.7	1.2	+1.5
		KW2008-003	3.8	2.8	+1.0
		KW2013007	4.1	2.3	+1.8
		MN14-07-17	5.3	3.8	+1.5
	Adult	AK2014085	15.9*	9.4**	+6.5

Differences between inner and outer muscle sites were significant; Using a paired t test; t = 4.020, d.f. = 4, p = 0.016. Note data on the adult humpback was not included in this analysis.

*Taken from Helbo etal.[48]: Muscle site presumed, but not confirmed

** Muscle sample site classification as outer based on physical appearance of the muscle tissue.

Anecdotally, the humpback whale adult tissue sample provided for this study (AK2014085) came from a region within the vicinity of the dorsal fin (Site b—Fig 1) however in this sample, the muscle tissue occurred in small bundles and had a bubbled and loose appearance. This is characteristic of the fascia boundary to the blubber (J. Rice; personal observation), suggesting that the tissue was peripheral muscle tissue. Comparing this value to the current published estimate of adult humpback whale Mb levels [48], this value was considerably lower than the current estimate (See Table 3). The single muscle sample used for the previously published estimate did not come from a known sampling site; however, the authors could confirm that the appearance was typical of interior portions of the muscle (S. Helbo; personal communication).

### 4. Early ontogeny of myoglobin levels in mysticete calves

**Fig 3:**
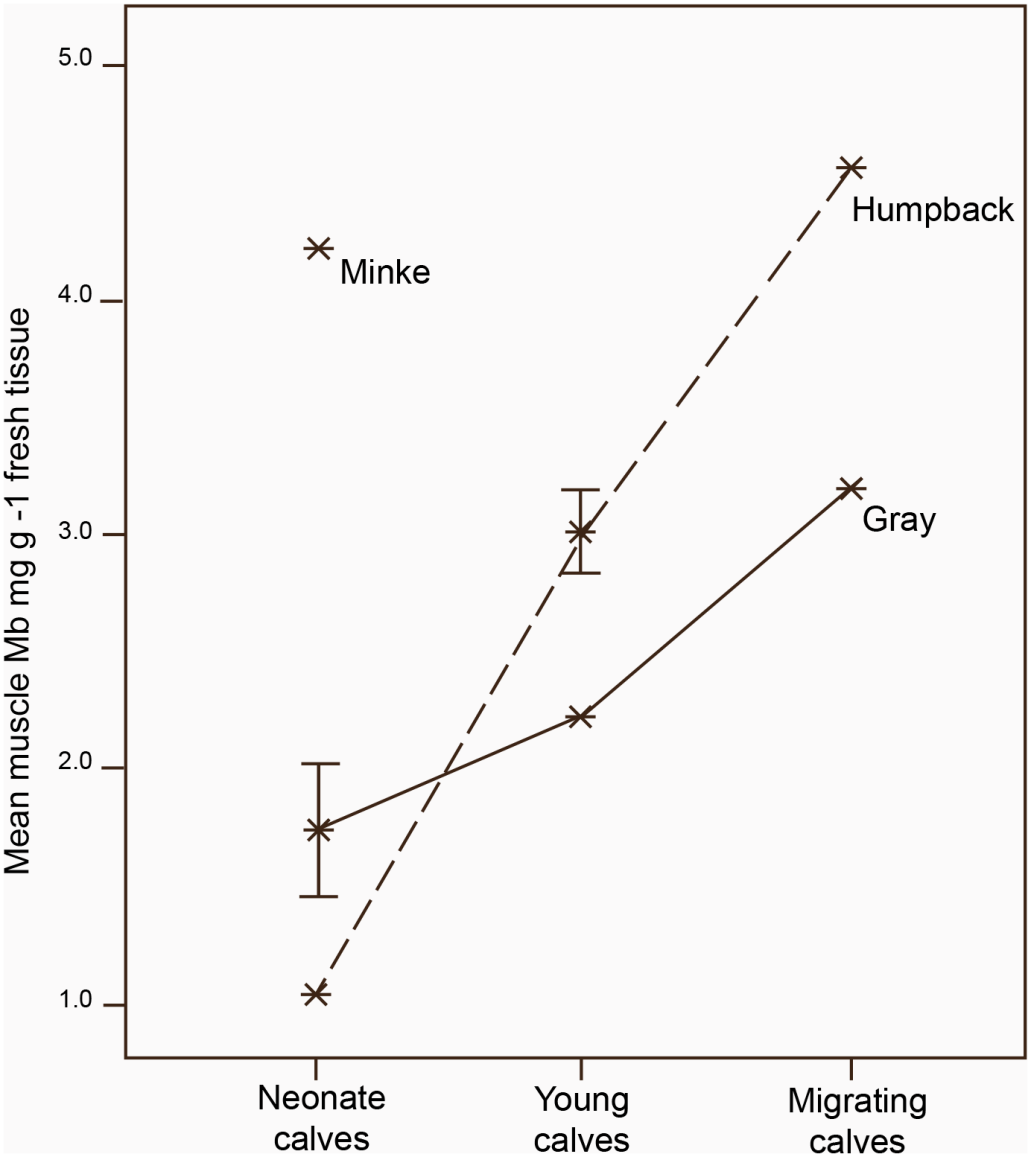
Early ontogeny of Mb levels in three species of mysticetes. Calf age classes based on growth curves described by ^a^ [44], ^b^ [45] and ^c^ [46], along with additional information from the field site of the necropsy. Estimated ages: Neonates < 2 weeks, young calves between 2 weeks to 3 months and stranded in breeding areas, migrating calves between 3 to 5 months and stranded in migratory corridors. Levels of Mb in the neonate minke whale lay beyond the upper limits of the 95% confidence interval for mean Mb levels in neonate humpback and gray whale calves (4.2 mg Mb g^-1^ vs. 0.0<1.5<3.0 mg Mb g^-1^). Levels of Mb rose faster in humpback vs. gray whale calves between the neonate, young and migratory age classes (for humpback whales β = 0.98 and for gray whales β = 0.41). Error bars indicate S.D.

In total, using data from this study along with data available in the literature (See S2 Table; n = 2 gray whale calves [3,6,49]), details of Mb levels in 12 mysticete calves were compiled. Ages ranged from neonates (estimated age < 2 weeks) to migrating calves (estimated age 3–5 months). To examine rates of change of Mb levels within this subset, a general linear model (GLM) was constructed, using muscular Mb as the dependent; relative calf age (classified as neonate, young or migrating calf) and species were included as fixed factors. Results indicated that Mb levels varied significantly between relative calf age cohorts and between species (for relative calf age; ANOVA F_2_ = 21.919, p = 0.002, for species; F_2_ = 10.281, p = 0.006). Using all three species, the interaction between species and age was not significant (ANOVA F_4_ = 3.938, p = 0.094). Unfortunately, only a single minke whale calf was included in the study, so to further investigate differences in rate of accumulation of Mb, the analysis was limited to a comparison of humpback and gray whale calves. Assigning approximate ages to the relative calf age classes (for neonates age = 0 .5 months, for young calves, approximate age = 1.5 months and for migrating calves, approximate age = 4 months) and comparing rates of change of muscular Mb levels in humpback and gray whale calves, the rate of increase in Mb levels was faster in humpback vs. gray whale calves (Fig 3; for humpback whales β = 0.98 and for gray whales β = 0.41). A GLM including mean Mb as the dependent, relative age in months as a covariate and species as a fixed factor suggested a significant interaction between species and relative calf age (ANOVA F_1_ = 14.903, p = 0.002) for humpback vs. gray whale calves over this period.

Examining differences in calf Mb levels between relative age classes using confidence intervals provides similar inferences. Comparing Mb levels in neonates, while differences between the humpback and the gray whale neonates were marginal (see Table 1), the single value for the neonate minke whale calf Mb level (Mb = 4.2 mg Mb g^1^) fell above the upper limits of the 95% C.I. for the mean muscular Mb for humpback and gray neonates combined (95% CI for the mean muscular Mb for neonate humpback and gray whale neonate calves; 0.0<1.5<3.0 mg Mb g^-1^), inferring a significant difference in Mb levels between minke vs. humpback and gray whale neonates. Looking at changes across this time period, in humpback whale calves, the mean Mb level for the migrating humpback whale calf fell above the 95% CI for the mean Mb for breeding ground (neonate and young) humpback calves (4.6 mg Mb g^1^ vs. 1.8<2.3<3.6 mg Mb g^1^), inferring that there was significant increase in Mb levels between the breeding grounds and early migration in humpback whale calves. By comparison, in the gray whale calves, the estimate for Mb levels in the migrating calf fell within the 95% CI for the mean Mb for breeding ground gray whale calves (3.2 mg Mb g^1^ vs. 0.5<1.9<3.2 mg Mb g^1^).

Relevant life history details for the three focal species over this period were compiled to review the degree of variability in early activity levels and other key developmental features, such as growth rates and length of lactation periods (see Table 4). Looking comparatively, minke whale calves are likely the most mobile of the three species in terms of swimming speeds [37], while humpback calves engage in frequent surface activity, such as extended sequences of breaching [50] and gray whale calves are typically quiescent during early development [51, 52]. Within the three focal species, relative growth rates are highest in gray whale calves [44], and lactation periods vary from 4–6 months in minke whale calves [44] to 10–12 months in humpback whale calves [34].

**Table 4:** Early life history traits for three species of mysticete calves.

Species	Birth length (m); birth mass (kg) ^a^. (Adult length; mass)	Activity levels in breeding regions	Growth rates Estimated daily rates ^a^ / comparative daily length gain*	Percentage fat in milk ^b^	Timing of weaning
Minke Whale *Balaenoptera acutorostrata*	2.4 m; 200 kg (Adult length 8.8 m / 9000 kg) ^a^	No observations from breeding areas. Typical swimming speeds 15–20 km/h ^c^	1.00 cm day^-1^ 1.13 x 10^−3^	30% at age 4–5 months	Prior to arrival in feeding areas ^a^
Humpback whale *Megaptera novaeangliae*	4.3 m; 2000 kg (Adult length 14 m / 33000 kg) ^d^	Young calves highly active, breaching sequences common throughout the breeding season ^e^	1.45 cm day ^-1^ 1.06 x 10^−3^	44% at age 4–7 months	Late season in feeding areas/ early season in breeding areas ^f^
Gray whale *Eschrichtius robustus*	4.7 m; 1200 kg (Adult length 13.5 m / 31,500 kg) ^g^	Calves quiescent, breaching rarely seen ^h^	1.71 cm day ^-1^ 1.2 x 10^−3^	53% at age 6–7 months	Within feeding areas ^j^

References: ^a^ [44], ^b^ [27], ^c^ [37], ^d^ [34] ^e^ [50] ^f^ [34], ^g^ [35] ^h, j^ [51,52].

* Calculated as a proportion of the final adult length

## Discussion

This study provides the first outlines of the developmental trajectories of muscular Mb stores for three species of mysticetes. As muscular Mb levels potentially dictate the aerobic dive limit in mysticetes, these trajectories may influence a wide range of related aspects of developmental and life history traits.

In the three species of mysticetes included in this study, the development of muscular Mb stores was protracted. Calves exhibited notably low levels of Mb and weaned juveniles carried only a portion of their eventual adult complement. Levels of Mb varied between and within muscles, with differences most pronounced in younger animals. Comparing the three species of mysticete calves included in this study, early levels and rates of accumulation of muscular Mb varied considerably between species. Comparing these differences to differences in life histories suggests that high levels of activity during early development may enhance the development of Mb stores during this period.

One of the main challenges inherent in this study is the small sample size, reflecting the challenges of obtaining usable tissue samples from stranded mysticetes. Notwithstanding, the sample includes three different species and three different age cohorts. Currently, available data on Mb levels in mysticetes are extremely limited and as a consequence, very early assessments of Mb levels from single animals (e.g. [53,54]) or theoretical, calculated estimates of myoglobin levels based on respiratory patterns (e.g. [55]) have been used in a wide range of comparative studies on the evolution, energetics and physiology of diving in mysticetes (e.g. [49,56–58]). This study therefore adds new and useful information to the limited dataset documenting mysticete Mb levels.

### Levels of Mb in adult mysticetes

Comparing the results of this study to previous reports, the minke whale estimate provided here is substantially higher than previously reported (24.2 (22.1–26.4) mg Mb g^-1^ vs. 7 mg Mb g^-1^ [48]. In the previous study, researchers reported the presence of an unexplained precipitate during the assay and suggested that this may have influenced the results. In this study, consistent results were obtained from a range of different muscles and from two different adult minke whales. Consequently, the results from this study would seem to be the more robust estimate.

The humpback whale estimate obtained in this study is considerably lower than the previously published estimate (9.4 mg Mb g^-1^ vs. 15.9 mg Mb g^-1^ [48]). Here, a feasible explanation may be based on field necropsy notes and the appearance of the tissue. The tissue sample used in this study had a bubbled, loose appearance, characteristic of peripheral tissue close to the fascia boundary to the blubber. The tissue sample used in the original study did not exhibit these characteristics (S Helbo; pers. comm.) Presuming that this tissue was taken from the interior regions of the muscle, this suggests that a pronounced gradient in Mb may exist within the muscle. The relatively high variability in gray whale adult Mb levels (9.2–16.3 mg Mb g^-1^) recorded in this study also infers that there may be a high degree of intra-muscular variability in Mb levels in mature mysticetes. To adequately assess intra-muscular variability in Mb levels, specific samples would need to be collected from a full range of known muscle sites during necropsies. In large mysticetes, this is certainly a challenge. However, as the results from such a study would provide valuable details for use in accurate, updated assessments of mysticete aerobic dive capacity, the investment of time and effort involved would certainly be well-justified.

Taken comparatively, differences in adult Mb between the three species included in this study fit well within the confines of previously reported trends in Mb levels across diving mammals with respect to increasing body mass. Essentially, as large body size confers increased metabolic efficiency this offsets the aerobic demands of extended dives [2, 49, 58]. Minke whales, the smallest species of the mysticetes, are considerably smaller than both humpback and gray whales (see table 4). Consequently, in order to sustain equivalent diving capabilities, minke whales would require higher Mb levels than seen in other, larger mysticetes, while the lower Mb levels reported here for humpback and gray whales may reflect the offset in the demands of aerobic dive capacity attributed to their larger body size.

### The developmental trajectory of Mb

Within the limited sample in the study, Mb levels increased with age and there were no indications of non-linear trends between specific age cohorts, as seen in other species of marine mammals such as Weddell seals [9]. Mysticete calf Mb levels ranged from 17–22.8% of adult levels and juveniles levels ranged between 28–60% of adult levels. As aerobic dive capacity tracks Mb levels [4], these low Mb levels likely contribute to the reduced dive times reported in younger mysticetes [46,50].

Ecologically, the consequences of reduced dive capacity are far-reaching: Predation risks increase [37,59] and increases in surfacing frequency and persistence raise the energetic costs of travel [60–62]. Additionally, foraging success for maternal females with dependent calves is impacted, due to the competing needs of offspring vigilance vs. diving [42]. These constraints persist through the juvenile phase, when constrained aerobic dive capacity would similarly increase travel costs and reduce foraging success [63].

Interestingly, the behavioral ecology of mysticetes includes a range of compensatory adaptive traits that offset these challenges. Predation risks are postponed as these three species of mysticetes are seasonal migrants and relocate to regions beyond the range of their key predator, the transient Orca, during the earliest periods of calf development. During travel, calves draft alongside maternal females [50,64] which provides hydrodynamic lift and reduces the energetic costs of travel [65]. Additionally, as capital breeders, maternal females use stored energy reserves during early calf development and maternal foraging is delayed until the latter periods of calf development in humpback and gray whales or until after weaning in minke whales. Furthermore, in juveniles, high levels of juvenile site fidelity to maternal feeding grounds [66,67] mean that newly weaned and juvenile whales benefit from maternal experience in prey choice and selection of foraging habitat during periods of limited dive capacity. Even key aspects of the juvenile mysticete morphology, specifically the shortened head and modified, smaller feeding apparatus, reduce the energetic costs of foraging during this life stage [63].

Taken cumulatively, these examples suggest that the limitations of the protracted ontogeny of the respiratory capacity through the calf and juvenile stages are integrated into the larger life history strategies and adaptive traits that characterize mysticetes. Notwithstanding though, in many ways this further underscores the costs and challenges associated with limited aerobic capacity during early development. Understanding the physiological mechanisms that may drive development of Mb in mysticetes provides the opportunity for further insight into this key aspect of ontogeny in young mysticetes.

At the molecular level, three factors are currently recognized as drivers in the development of Mb stores during early ontogeny, namely hypoxia, lipid supplementation and exercise, [24–26,29]. As all mysticetes are born into water and maintain an entirely aquatic lifestyle, exposure to hypoxia immediately following parturition is ubiquitous among mysticetes. Based on analysis of cetacean milk samples, while differences in milk composition are seen towards the later stages of lactation, proportions of lipid in the early diet are also consistent in the three species included here [27]. However, levels of exercise vary interspecifically between the three focal species and cumulatively, a range of results from this study suggest that exercise assumes a driving role in the development of muscular Mb stores in young mysticetes.

Firstly, comparing Mb levels in different muscles of the same animal, in young animals, levels of Mb were greater in the epaxial vs. hypaxial portions of the muscle. Both of these muscles play a role in providing propulsion during swimming; the epaxial muscles provide the upstroke of the fluke and the hypaxial muscles are responsible for the downstroke [68]. Although equivalent levels of propulsion are provided during the up- and down stroke [69], drag is greater during the upstroke vs. the downstroke [70], so levels of exertion are likely higher during the upstroke [71]. Higher levels of Mb in the epaxial muscles therefore correspond to higher levels of exertion during swimming. In marine mammals, the costs of swimming scales inversely with body size [38]. Despite the benefits of drafting alongside the mother during swimming [65], high fluke beat rates compared to conspecific adults are a distinct characteristic of young cetaceans [65,72] and would potentially amplify differences in exertion between the epaxial and hypaxial muscles. Looking at these results collectively, as differences were less pronounced in the adults included in this study, a tentative inference could be that heterogeneity in Mb levels between muscle sites in mysticetes is an ontogenetic phenomenon reflecting the exertion of swimming during early development. As the costs of swimming diminish with increasing body size [38], the difference may then become less pronounced as whales mature.

A second connection between levels of exercise and levels of Mb emerges when comparing samples from different depths within the same muscle site. In this study, confirmed site-specific samples were only available from humpback whale calves (n = 5). Within this group, Mb levels were significantly higher in the deepest regions of the epaxial muscle compared to outer regions of the same muscle. The inner portions of the muscle lying closest to the skeleton are under the greatest stress and exertion during exercise [43], so muscle activity would be elevated relative to adjacent areas. Higher Mb levels in this area therefore correspond to the levels of exertion and exercise in the inner vs. the outer portions of the same muscle.

Finally, reviewing interspecific differences in temporal changes in Mb levels during early development, differing rates of development of Mb stores correspond to interspecific differences in levels of activity and exercise between the three mysticete species included in this study. In minke whales, although reports of female-calf behavior are exceptionally sparse, adult minke whales are remarkably fast swimmers [37]. Assuming that maternal females maintain relatively fast swimming speeds and calves maintain the close proximity and consistent activity that is typical across cetacea [33], young minke whales would be exposed to high levels of exercise from birth, which could contribute to elevated Mb levels. Notwithstanding, given the elevated Mb levels in the minke neonate in comparison to the other two species, additional mechanisms that may somehow prime the muscles prior to parturition may be at play, as suggested by other researchers [15]. Both the diminutive body size of neonate minke whales in comparison to other mysticete calves, and the comparatively short lactation period relative to other mysticetes, correspond to life-history trends seen in species of odontocetes that exhibit very early maturation of muscular Mb stores.

Comparing humpback and gray whale calves, in both cases, Mb levels in the youngest calves were extremely low in comparison to the minke calf. As calves matured, Mb levels increased, however rates of increase were significantly faster for humpback whale calves in comparison to gray whale calves (see Fig 3). By the beginning of the natal migration, the migrating humpback whale calf had significantly higher levels of muscular Mb than seen in breeding regions while gains in gray whale calves over this period were marginal.

Levels of exercise in young humpback and gray whales differ markedly during this period. During their time in breeding regions, gray whale calves are described as quiescent and aerial behaviors such as breaching, though seen, are very rare [51,52]. In contrast, humpback whale calves are highly active, especially during the earliest periods of development [50,73]. Extended sequences of breaching are common and frequently include 30 or more consecutive breaches [50,73]. While single breaches may not be energetically costly, sequences of breaching behavior carry high energetic costs [74] and the social contexts that may justify the costs of this behavior in adults are not relevant to young calves [74,75]. Previous researchers focusing on early development in mysticete calves have speculated on a possible connection between exercise during early development and the ontogeny of the respiratory capacity [52,76]. With the mechanism that links exercise to the production of Mb via the calcium-calcineurin pathway now established, this potentially justifies the diversion of finite energy reserves to the support high levels of activity that optimize the rate of development of the respiratory capacity in young humpback whale calves. Notably, growth rates in gray whale calves exceed growth rates in humpback calves during this period, despite the overall smaller size of gray whales [44]. Implicitly, this suggests a trade-off between optimal growth and exercise during this crucial developmental period. Potentially this trade-off plays out differently in these two mysticetes, perhaps reflecting the differing threats and pressures of the impending natal migration.

In summary, this study provides new updates on muscular Mb levels in a range of mysticete adults and provides the first outlines of key factors that may drive the ontogeny of the respiratory capacity of young mysticetes. Still, this study leaves many questions unanswered. For example, the ability of mysticetes to utilize alternate respiratory pathways and support anaerobic respiration during extended dives has yet to be established. Additionally, proportions and changes in types of muscle tissue (i.e. type 1 vs type 2, slow vs fast twitch tissue) are also un-documented at this point. Based on recent simulations of mysticete dive capacities [63] these factors may prove to be very pertinent in discussions of breath-hold capacity and the associated foraging tactics used by both young and mature mysticetes. Further studies in this field will clearly be required for a full understanding of mysticete respiratory capacity. For now, as marine resources enter a period of unprecedented change [77,78], these results bring focus to the physiological constraints on diving and foraging in young and maturing mysticetes, allowing informed insight into their resilience to the impending challenges facing marine fauna.

## Supporting Information

S1 Table Stranding details for sources of tissue samples. (DOCX)

S2 Table Mysticete muscular Mb levels sourced from the literature. (DOCX)
