# Supplementary material for: Tracking the Development of Muscular Myoglobin Stores in Mysticete Calves
Source: PLoS One. 2016 Jan 20;11(1):e0145893. doi: 10.1371/journal.pone.0145893 (PMC4720374; doi:10.1371/journal.pone.0145893)
Supplement: S1 Table — (DOCX) [file pone.0145893.s001.docx]

Table S1: Stranding details for sources of tissue samples.

| Species | Age class | Date of stranding | Condition | Length  (cm) | Tissue  Source | Notes |
| --- | --- | --- | --- | --- | --- | --- |
| Gray whale  (HMSC04-C1) | Calf | 1/2004 | Moderate decomposition | 436 | OMMSN | Male, scars on pectoral fins and in mouth indicate entanglement |
| Gray whale  (HMSC06-05-14-Er) | Calf | 5/2006 | Fresh dead | 584 | OMMSN | Male, found live, entangled in crab pot, subsequently died |
| Gray whale  (HMSC15-05-15-Er) | Juvenile | 5/2015 | Fresh dead | 777 | OMMSN | Clear signs of orca predation: multiple deep tooth rake marks, bites, widespread bruising to the blubber |
| Gray whale  (HMSC06-05-24-Er) | Adult | 5/ 2006 | Moderate decomposition | 1282 | OMMSN | Female |
| Gray whale  (HMSC09-04-09-Er) | Adult | 4/2009 | Fresh dead | 1311 | OMMSN | Female, died on beach, large cauliflower like growth on one ovary and very emaciated |
| Humpback whale  (KW2013012) | Calf | 4/ 2013 | Fresh dead | 399 | HPUSP | Carcass lost to sharks as it was being towed to the necropsy site |
| Humpback whale  (KW200801) | Calf | 2/ 2008 | Live stranding. | 410 | HPUSP | Live stranding on beach. Died same day. Necropsy performed immediately |
| Humpback whale  (KW2013010) | Calf | 3/ 2013 | Fresh dead | 432 | HPUSP | Reported stranded then dead on beach. Necropsy done the next day |
| Humpback whale  (KW2013002) | Calf | 1/2013 | Fresh dead | 457 | HPUSP | Live stranding, died on beach. Umbilicus slightly healed. |
| Humpback whale  (KW2013007) | Calf | 3/ 2013 | Fresh dead | 480 | HPUSP | Male. Fresh dead, found on beach. |
| Humpback whale  (KW200901) | Calf | 12/ 2009 | Fresh dead | 518 | HPUSP | Found floating at sea, but fresh dead. No signs of decomposition or sun damage |
| Humpback whale  (Mn14-07-17) | Calf | 7/2014 | Fresh dead | 650 | OMMSN | Male found stranded on beach, fresh but deceased. No information on cause of death. |
| Humpback whale  (HMSC10-09-19-Mn) | Juvenile | 9/ 2010 | Fresh dead | 1055 | OMMSN | Male, Signs of orca attack, hyoid bone dislocated and loose, only partial remains of tongue |
| Humpback whale  (AK2014085) | Adult | 7/2014 | Fresh dead | 1462 | AKSN | Female - Ship strike, clear signs of blunt force trauma |
| Minke Whale  (HMSC04-C5) | Calf | 4/ 2004 | Fresh dead | 240 | OMMSN | No details provided |
| Minke whale  (IFAW 130ba) | Juvenile | 7/2014 | Fresh dead | 505 | IFAW | Male, stranded then refloated. Found dead at sea the following day |
| Minke whale  (IFAW 131ba) | Adult | 7/2014 | Fresh dead | 755 | IFAW | Male, first sighted alive but stranded in heavy surf. Died on the beach. |
| Minke whale  (IFAW 100ba) | Adult | 7/2014 | Fresh dead | 790 | IFAW | Female, originally sighted dead and entangled in lobster trap gear. |
