# Supplementary material for: Tracking the Development of Muscular Myoglobin Stores in Mysticete Calves
Source: PLoS One. 2016 Jan 20;11(1):e0145893. doi: 10.1371/journal.pone.0145893 (PMC4720374; doi:10.1371/journal.pone.0145893)
Supplement: S2 Table — (DOCX) [file pone.0145893.s002.docx]

Table S2: Mysticete muscular Mb levels sourced from the literature

| Species | Age class | Body Length  (cm) | Source |
| --- | --- | --- | --- |
| Gray whale | Neonate | Unknown | From [6]; calf classified as a neonate. No additional details provided. Used in this study. |
| Gray whale | Neonate | Estimated:  289 cm | From [3]; calf classified as a neonate based on field notes, including body weight (900kg), location of stranding (San Diego, CA) and timing (1/11/84) Details provided by M. Castellini. Used in this study. Body length estimate based on [78]. Used in this study. |
| Humpback whale | Adult | Unknown | From [48]. No sample details available beyond classification as an adult. Appearance of tissue confirmed as typical for adult muscle tissue by S. Helbo. Used in this study. |
| Minke Whale | Adult | Unknown | From [48]. The authors report that the appearance of an unusual precipitate during the experimental procedure, therefore this estimate was not used in this study. |
